# Supplementary material for: Relationship between evacuation after the Great East Japan Earthquake and new-onset hyperuricemia: A 7-year prospective longitudinal study of the Fukushima Health Management Survey
Source: PLoS One. 2023 Oct 26;18(10):e0293459. doi: 10.1371/journal.pone.0293459 (PMC10602330; doi:10.1371/journal.pone.0293459)
Supplement: S1 Table — The values in the table indicate the average value (standard deviation) or the percentage. Fasting blood glucose, triglycerides, AST, ALT, and γ-GT are reported as the median (25–75% percentile). BMI: body mass index, HbA1c: hemoglobin A1c, HDL-C: high-density lipoprotein cholesterol, LDL-C: low-density lipoprotein cholesterol, AST: aspartate aminotransferase, ALT: alanine aminotransferase, γ-GT: γ-glutamyl transpeptidase, eGFR: estimated glomerular filtration rate. (DOCX) [file pone.0293459.s001.docx]

Table S1. Clinical and biochemical characteristics of the 18,140 participants stratified by sex and the development of hyperuricemia

|  | Men | | |  | Women | | |
| --- | --- | --- | --- | --- | --- | --- | --- |
|  | Hyperuricemia (uric acid > 7 mg/dL) | | p |  | Hyperuricemia (uric acid > 6 mg/dL) | | p |
|  | Not incident | Incident |  |  | Not incident | Incident |  |
| n (%) | 5,353 (76.9) | 1,608 (23.1) |  |  | 9,791 (87.6) | 1,388 (12.4) |  |
| Age (years) | 61.1 (8.6) | 61.4 (8.5) | 0.193 |  | 59.0 (9.1) | 60.7 (8.7) | <0.001 |
| BMI (kg/m^2^) | 24.2 (3.0) | 24.8 (3.1) | <0.001 |  | 23.1 (3.4) | 24.9 (3.8) | <0.001 |
| BMI ≥ 25 kg/m^2^ (%) | 37.6 | 44.3 | <0.001 |  | 26.3 | 44.3 | <0.001 |
| Systolic blood pressure (mmHg) | 132 (15) | 136 (15) | <0.001 |  | 128 (16) | 133 (16) | <0.001 |
| Diastolic blood pressure (mmHg) | 81 (10) | 82 (10) | 0.001 |  | 77 (10) | 80 (10) | <0.001 |
| Hypertension (%) | 54.8 | 65.5 | <0.001 |  | 40.9 | 60.2 | <0.001 |
| HbA1c (%) | 5.6 (0.8) | 5.6 (0.8) | 0.027 |  | 5.5 (0.6) | 5.6 (0.7) | <0.001 |
| Fasting blood glucose (mg/dL) | 100 (93-111) | 100 (93-109) | 0.328 |  | 94 (89-101) | 97 (90-105) | <0.001 |
| Diabetes (%) | 18.0 | 14.4 | <0.001 |  | 7.3 | 10.2 | <0.001 |
| HDL-C (mg/dL) | 56 (14) | 55 (14) | <0.001 |  | 65 (15) | 61 (15) | <0.001 |
| LDL-C (mg/dL) | 124 (32) | 121 (32) | 0.002 |  | 130 (32) | 131 (33) | 0.535 |
| Triglycerides (mg/dL) | 104 (75-147) | 114 (80-168) | <0.001 |  | 88 (65-121) | 110 (80-148) | <0.001 |
| Dyslipidemia (%) | 56.3 | 59.1 | 0.043 |  | 57.3 | 66.2 | <0.001 |
| AST (U/L) | 24 (20-29) | 25 (21-30) | <0.001 |  | 21 (18-25) | 22 (19-27) | <0.001 |
| ALT (U/L) | 22 (16-31) | 23 (17-33) | 0.001 |  | 16 (13-22) | 19 (14-27) | <0.001 |
| γ-GT (U/L) | 32 (21-54) | 39 (26-63) | <0.001 |  | 18 (14-26) | 22 (16-33) | <0.001 |
| Abnormal liver function (%) | 43.1 | 50.7 | <0.001 |  | 17.4 | 28.2 | <0.001 |
| eGFR (mL/min/1.73 m^2^) | 74.7 (13.4) | 71.4 (13.1) | <0.001 |  | 75.2 (12.5) | 69.8 (13.4) | <0.001 |
| Abnormal renal function (%) | 11.6 | 17.6 | <0.001 |  | 9.0 | 20.9 | <0.001 |
| Uric acid (mg/dL) | 5.2 (1.0) | 6.2 (0.7) | <0.001 |  | 4.1 (0.8) | 5.2 (0.6) | <0.001 |

The values in the table indicate the average value (standard deviation) or the percentage. Fasting blood glucose, triglycerides, AST, ALT, and γ-GT are reported as the median (25-75% percentile). BMI: body mass index, HbA1c: hemoglobin A1c, HDL-C: high-density lipoprotein cholesterol, LDL-C: low-density lipoprotein cholesterol, AST: aspartate aminotransferase, ALT: alanine aminotransferase, γ-GT: γ-glutamyl transpeptidase, eGFR: estimated glomerular filtration rate.
