# Supplementary material for: Relationship between evacuation after the Great East Japan Earthquake and new-onset hyperuricemia: A 7-year prospective longitudinal study of the Fukushima Health Management Survey
Source: PLoS One. 2023 Oct 26;18(10):e0293459. doi: 10.1371/journal.pone.0293459 (PMC10602330; doi:10.1371/journal.pone.0293459)
Supplement: S2 Table — The values in the table indicate the number (percentage). K6: Kessler 6-item scale, PTSD: Post-traumatic stress disorder, PCL: Post-traumatic Stress Disorder Checklist. (DOCX) [file pone.0293459.s002.docx]

Table S2. Lifestyle characteristics of the 18,140 participants stratified by sex and the development of hyperuricemia

|  |  | Men | | |  | Women | | |
| --- | --- | --- | --- | --- | --- | --- | --- | --- |
|  |  | Hyperuricemia (uric acid > 7 mg/dL) | | p |  | Hyperuricemia (uric acid > 6 mg/dL) | | p |
|  |  | Not incident | Incident |  |  | Not incident | Incident |  |
| Evacuation experience | Yes | 2,390 (44.7) | 841 (52.3) | <0.001 |  | 4,428 (45.2) | 746 (53.8) | <0.001 |
| Smoking status | Never smoker | 1,455 (27.5) | 374 (23.5) | 0.006 |  | 8,319 (88.5) | 1,113 (84.3) | <0.001 |
|  | Quit smoking | 2,406 (45.5) | 770 (48.3) |  |  | 532 (5.7) | 106 (8.0) |  |
|  | Current smoker | 1,428 (27.0) | 451 (28.3) |  |  | 549 (5.8) | 101 (7.7) |  |
| Drinking status | Never drinker | 1,375 (25.8) | 312 (19.5) | <0.001 |  | 6,793 (71.2) | 859 (63.9) | <0.001 |
|  | Quit drinking | 253 (4.8) | 63 (3.9) |  |  | 87 (0.9) | 26 (1.9) |  |
|  | < 44 g/day | 2,538 (47.7) | 740 (46.1) |  |  | 2,499 (26.2) | 403 (30.0) |  |
|  | ≥ 44 g/day | 1,156 (21.7) | 489 (30.5) |  |  | 163 (1.7) | 57 (4.2) |  |
| Sleep satisfaction | Satisfied | 1,818 (41.1) | 550 (41.2) | 1.000 |  | 489 (30.5) | 294 (26.2) | 0.473 |
|  | Slightly dissatisfied | 1,867(42.2) | 563 (42.1) |  |  | 3,984 (49.4) | 540 (48.2) |  |
|  | Very dissatisfied | 589 (13.3) | 177 (13.3) |  |  | 1,455 (18.1) | 218 (19.5) |  |
|  | Unable to sleep | 152 (3.4) | 46 (3.4) |  |  | 439 (5.5) | 69 (6.2) |  |
| Physical activity | Every day | 1,016 (19.3) | 327 (20.7) | 0.127 |  | 1,370 (14.3) | 189 (13.8) | 0.405 |
|  | 2-4 times a week | 1,198 (22.8) | 389 (24.6) |  |  | 2,370 (24.7) | 352 (25.8) |  |
|  | Once a week | 820 (15.6) | 220 (13.9) |  |  | 1,444 (15.1) | 223 (16.3) |  |
|  | None | 2,222 (42.3) | 646 (40.8) |  |  | 4,409 (46.0) | 602 (44.1) |  |
| Changes in work situation | Yes | 2,998 (57.5) | 955 (61.3) | 0.008 |  | 5,250 (57.3) | 714 (55.6) | 0.251 |
| Unemployment experience | Yes | 1,041 (23.7) | 366 (27.9) | 0.002 |  | 2,370 (28.3) | 310 (27.4) | 0.504 |
| Tsunami experience | Yes | 1,247 (23.3) | 369 (23.0) | 0.772 |  | 1,685 (17.2) | 211 (15.2) | 0.062 |
| Nuclear accident experience | Yes | 2,985 (55.8) | 939 (58.4) | 0.062 |  | 5,174 (52.8) | 768 (55.3) | 0.082 |
| Psychological distress | K6 ≥ 13 | 542 (11.2) | 175 (12.1) | 0.370 |  | 1,525 (17.2) | 230 (18.6) | 0.224 |
| PTSD | PCL ≥ 44 | 895 (18.2) | 274 (18.7) | 0.695 |  | 2,194 (24.4) | 342 (27.1) | 0.037 |

The values in the table indicate the number (percentage).

K6: Kessler 6-item scale, PTSD: Post-traumatic stress disorder, PCL: Post-traumatic Stress Disorder Checklist.
