# Supplementary material for: Relationship between evacuation after the Great East Japan Earthquake and new-onset hyperuricemia: A 7-year prospective longitudinal study of the Fukushima Health Management Survey
Source: PLoS One. 2023 Oct 26;18(10):e0293459. doi: 10.1371/journal.pone.0293459 (PMC10602330; doi:10.1371/journal.pone.0293459)
Supplement: S3 Table — Model 1: adjusted for age, BMI, systolic blood pressure, fasting blood glucose, triglycerides, eGFR, smoking status, drinking status, and unemployment experience. Model 2: adjusted for Model 1 plus sleep dissatisfaction, physical activity, tsunami experience, nuclear accident experience and, post-traumatic stress disorder. HR: hazard ratio, CI: confidence interval, BMI: body mass index. (DOCX) [file pone.0293459.s003.docx]

Table S3. Hazard ratios of evacuation for the development of hyperuricemia (levels higher than 7 mg/dL) in 6,961 men, according to baseline variates

|  |  | Model 1 | |  | Model 2 | |
| --- | --- | --- | --- | --- | --- | --- |
|  |  | HR (95% CI) | p |  | HR (95% CI) | p |
| Age | *≥ 65 years* | 1.18 (0.99-1.40) | 0.063 |  | 1.19 (1.00-1.42) | 0.052 |
|  | *< 65 years* | 1.07 (0.93-1.24) | 0.318 |  | 1.06 (0.92-1.22) | 0.422 |
| BMI | *≥ 25.0 kg/m^2^* | 1.09 (0.92-1.29) | 0.308 |  | 1.07 (0.91-1.27) | 0.415 |
|  | *< 25.0 kg/m^2^* | 1.16 (1.00-1.34) | 0.050 |  | 1.15 (0.99-1.33) | 0.071 |
| Smoking status | *Quit or current smoker* | 1.14 (1.00-1.29) | 0.046 |  | 1.13 (1.00-1.29) | 0.057 |
|  | *Never smoker* | 1.06 (0.85-0.32) | 0.622 |  | 1.06 (0.85-1.34) | 0.591 |
| Drinking status | *Quit or current drinker* | 1.18 (1.04-1.33) | 0.008 |  | 1.17 (1.03-1.32) | 0.013 |
|  | *Never drinker* | 0.94 (0.73-1.21) | 0.631 |  | 0.93 (0.72-1.21) | 0.593 |
| Anti-hypertensive agents | *Yes* | 1.20 (1.02-1.40) | 0.025 |  | 1.22 (1.04-1.43) | 0.017 |
|  | *No* | 1.04 (0.89-1.21) | 0.643 |  | 1.02 (0.87-1.18) | 0.844 |
| Hypertension | *Yes* | 1.13 (0.99-1.29) | 0.074 |  | 1.14 (1.00-1.31) | 0.057 |
|  | *No* | 1.06 (0.88-1.28) | 0.522 |  | 1.03 (0.85-1.25) | 0.749 |
| Diabetes | *Yes* | 1.18 (0.88-1.57) | 0.274 |  | 1.18 (0.87-1.58) | 0.284 |
|  | *No* | 1.10 (0.98-1.24) | 0.104 |  | 1.10 (0.97-1.24) | 0.130 |
| Abnormal renal function | *Yes* | 1.24 (0.96-1.60) | 0.102 |  | 1.31 (1.01-1.70) | 0.046 |
|  | *No* | 1.09 (0.97-1.23) | 0.152 |  | 1.08 (0.95-1.22) | 0.225 |

Model 1: adjusted for age, BMI, systolic blood pressure, fasting blood glucose, triglycerides, eGFR, smoking status, drinking status, and unemployment experience. Model 2: adjusted for Model 1 plus sleep dissatisfaction, physical activity, tsunami experience, nuclear accident experience and, post-traumatic stress disorder. HR: hazard ratio, CI: confidence interval, BMI: body mass index.
