# Supplementary material for: Relationship between evacuation after the Great East Japan Earthquake and new-onset hyperuricemia: A 7-year prospective longitudinal study of the Fukushima Health Management Survey
Source: PLoS One. 2023 Oct 26;18(10):e0293459. doi: 10.1371/journal.pone.0293459 (PMC10602330; doi:10.1371/journal.pone.0293459)
Supplement: S4 Table — Model 1: adjusted for age, BMI, systolic blood pressure, fasting blood glucose, triglycerides, eGFR, smoking status, drinking status, and unemployment experience. Model 2: adjusted for Model 1 plus sleep dissatisfaction, physical activity, tsunami experience, nuclear accident experience and, post-traumatic stress disorder. HR: hazard ratio, CI: confidence interval, BMI: body mass index. (DOCX) [file pone.0293459.s004.docx]

Table S4. Hazard ratio of evacuation for the development of hyperuricemia (levels higher than 6 mg/dL) in 11,179 women, according to baseline variates

|  |  | Model 1 | |  | Model 2 | |
| --- | --- | --- | --- | --- | --- | --- |
|  |  | HR (95% CI) | p |  | HR (95% CI) | p |
| Age | *≥ 65 years* | 1.13 (0.93-1.37) | 0.209 |  | 1.13 (0.93-1.37) | 0.227 |
|  | *< 65 years* | 1.23 (1.06-1.43) | 0.006 |  | 1.20 (1.03-1.39) | 0.019 |
| BMI | *≥ 25.0 kg/m^2^* | 1.18 (0.99-1.40) | 0.064 |  | 1.16 (0.98-1.39) | 0.091 |
|  | *< 25.0 kg/m^2^* | 1.19 (1.02-1.39) | 0.028 |  | 1.18 (1.00-1.38) | 0.046 |
| Smoking status | *Quit or current smoker* | 1.13 (0.82-1.55) | 0.451 |  | 1.12 (0.81-1.54) | 0.506 |
|  | *Never smoker* | 1.23 (1.08-1.39) | 0.002 |  | 1.20 (1.05-1.37) | 0.007 |
| Drinking status | *Quit or current drinker* | 1.27 (1.04-1.54) | 0.019 |  | 1.24 (1.01-1.51) | 0.038 |
|  | *Never drinker* | 1.19 (1.03-1.38) | 0.022 |  | 1.17 (1.01-1.36) | 0.042 |
| Anti-hypertensive agents | *Yes* | 1.14 (0.96-1.35) | 0.130 |  | 1.14 (0.96-1.35) | 0.143 |
|  | *No* | 1.23 (1.05-1.44) | 0.013 |  | 1.20 (1.02-1.41) | 0.031 |
| Hypertension | *Yes* | 1.17 (1.01-1.35) | 0.041 |  | 1.16 (1.00-1.35) | 0.050 |
|  | *No* | 1.21 (1.00-1.46) | 0.049 |  | 1.17 (0.96-1.41) | 0.117 |
| Diabetes | *Yes* | 1.09 (0.76-1.57) | 0.652 |  | 1.03 (0.71-1.51) | 0.871 |
|  | *No* | 1.21 (1.07-1.37) | 0.003 |  | 1.20 (1.06-1.36) | 0.005 |
| Abnormal renal function | *Yes* | 0.97 (0.75-1.26) | 0.818 |  | 0.96 (0.74-1.26) | 0.775 |
|  | *No* | 1.23 (1.08-1.41) | 0.002 |  | 1.21 (1.06-1.38) | 0.005 |

Model 1: adjusted for age, BMI, systolic blood pressure, fasting blood glucose, triglycerides, eGFR, smoking status, drinking status, and unemployment experience. Model 2: adjusted for Model 1 plus sleep dissatisfaction, physical activity, tsunami experience, nuclear accident experience and, post-traumatic stress disorder. HR: hazard ratio, CI: confidence interval, BMI: body mass index.
